# Recent Developments in Aptamer-Based Sensors for Diagnostics

**DOI:** 10.3390/s24237432

**Published:** 2024-11-21

**Authors:** Muhammad Sheraz, Xiao-Feng Sun, Yongke Wang, Jiayi Chen, Le Sun

**Affiliations:** School of Chemistry and Chemical Engineering, Northwestern Polytechnical University, Xi’an 710072, China; sherazmuhammad@mail.nwpu.edu.cn (M.S.); shidaijiuji@mail.nwpu.edu.cn (Y.W.);

**Keywords:** chronic and non-communicable diseases, aptamer-based biosensors, SELEX, point-of-care diagnostics

## Abstract

Chronic and non-communicable diseases (NCDs) account for a large proportion of global disorders and mortality, posing significant burdens on healthcare systems. Early diagnosis and timely interference are critical for effective management and disease prevention. However, the traditional methods of diagnosis still suffer from high costs, time delays in processing, and infrastructure requirements that are usually unaffordable in resource-constrained settings. Aptamer-based biosensors have emerged as promising alternatives to offer enhanced specificity, stability, and cost-effectiveness for disease biomarker detection. The SELEX (Systematic Evolution of Ligands by Exponential Enrichment) methodology allows developing aptamers with high-affinity binding capabilities to a variety of targets, for instance proteins, cells, or even small molecules, hence rendering them suitable for NCD diagnosis. Aptasensors—recent developments in the electrochemical and optical dominion—offer much enhanced sensitivity, selectivity, and stability of detection across a diverse range of diseases from lung cancer and leukemia to diabetes and chronic respiratory disorders. This study provides a comprehensive review of progress in aptamer-based sensors, focusing on their role in point-of-care diagnostics and adaptability in a real-world environment with future directions in overcoming current limitations.

## 1. Introduction

**Figure 1:**
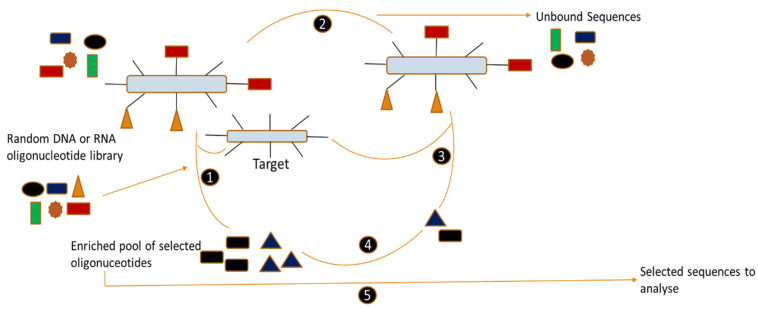
Schematic diagram of SELEX process showing the several stages taken by SELEX to produce aptamers. (1) Target incubation. (2) Dividing up. (3) Separation/elution. (4) PCR or RT-PCR amplification. (5) Cloning of chosen aptamer pool following the last SELEX phase [9].

Aptamers are short, single-stranded molecules made of DNA, RNA or modified nucleic acids that can bind to specific targets with high affinity and selectivity [1,2,3,4]. Aptamers generated via the systematic evolution of ligands by exponential enrichment (SELEX) process exhibit strong affinity and high specificity to target molecules [5]. Figure 1 is a schematic diagram of the SELEX process. Any SELEX procedure begins with a collection of a 10^13^–10^15^ synthesized DNA oligonucleotides library. During the first step, the library and the compound of interest are incubated together. Subsequently, a step called partitioning is used where non-binding oligonucleotides are partitioned away from the solution [6]. Aptamers are molecules that form a complex with their target, subsequently dissociating from it during the elution phase. Polymerase chain reaction (PCR) or reverse transcription polymerase chain reaction (RT-PCR) is then used in the amplification of the sequences depending on whether they are RNA or DNA [7]. Usually eight to fifteen rounds are performed in order to obtain a high-affinity aptamer [8].

In response to recent trends in infectious disease outbreaks, numerous aptamers have been developed to specifically recognize bacterial and viral targets. Over the past four years, considerable efforts have been made globally to isolate aptamers capable of binding to SARS-CoV-2. These aptamers are being evaluated for their potential as diagnostics [1,10,11]. Aptamers have been widely explored for diagnostics and the therapeutic intervention of various human ailments, including Alzheimer’s and cancer. However, clinical success has been limited, with only one aptamer, Pegaptanib (Macugen; Pfizer/Eyetech, New York, NY, USA), initially demonstrating efficacy. Pegaptanib was approved by the FDA in 2004 for treating age-related macular degeneration (AMD) [12] but was later discontinued as more effective treatments became available [13]. Recently, a second aptamer, Avacincaptad pegol (Izervay; Iveric Bio, Parsippany, NJ, USA/Asetlla, Tokyo, Japan), received approval in August 2023 for geography atrophy (GA) secondary to AMD [14]. Although further data are needed to evaluate its effectiveness compared to other GA treatments, Phase 3 clinical trials (GATHER1 and GATHER2) have shown that Avacincaptad pegol significantly slowed GA progression over months [14]. Chronic diseases remain a major global public health issue, ranking among the leading causes of illness and death. In the biomedical field, accurately and rapidly detecting disease biomarkers are essential for early diagnosis and health monitoring [15,16]. Trace levels of biomarkers within complex biological samples, such as blood, pose challenges that complicate disease diagnosis [17,18]. To address this, highly selective molecular recognition elements along with sensitive detection systems are needed. Aptamer-based biosensors have been developed to fulfill these requirements effectively [19]. 

## 2. Aptamers Versus Antibodies

Aptamers are advantageous due to their ease of production and scalability with significantly shorter production time than antibodies. Being chemically synthesized, aptamers offer high batch-to-batch consistency and greater design flexibility. Aptamers are thermally stable and maintain their structure and function across a broad temperature range, which eliminates the need for refrigeration during storage and transport. Additionally, aptamers can be reused in various applications, as they can be denatured with heat and renatured upon cooling [20]. Compared to antibodies, aptamers provide multiple benefits: they do not provoke immune responses, have broader targeting capabilities, and do not require animal use in production. Aptamers also show superior renal clearance and tissue penetrability, and their production can be rapidly scaled up [21,22,23,24,25]. A detailed comparison between antibodies and aptamers is presented in Table 1.

## 3. Aptamer-Based Biosensors

Aptamer-based biosensors utilize aptamers as recognition elements for the detection of a target, which is often coupled with a readable signal. Indeed, a wide range of aptasensors have been developed for the recognition and measurement of small analytes like toxins to large proteins [28,29,30,31,32]. These sensors have also been developed for use in a wide range of format types, including colorimetric assays utilized in medical testing [33] and lateral flow assays (LFAs) for diagnostic purposes [34,35]. When an aptamer binds with its target, a wide range of signals may be generated, including but not limited to optical, colorimetric, or electrochemical, which will be detected and measured [36,37,38]. 

Aptamers can withstand rigorous cleaning protocols, making them suitable for reuse in multiple measurements [39]. Aptasensors are a subclass of biosensors that utilize aptamers for the highly sensitive detection of various biomolecules [40,41]. Aptamers are also employed in biosensors as identifying components [42,43]. They can be put on the biosensor surface with a higher density because they are 10–100 times smaller than antibodies. A smaller volume of the test sample is needed for aptamer-based biosensors, and they can be reused repeatedly without losing sensitivity [44,45,46]. Aptamers are commonly used in biosensors because they are easy to design and can bind to specific targets by forming stable tertiary structures [47]. 

### 3.1. Electrochemical Aptasensors

**Figure 2:**
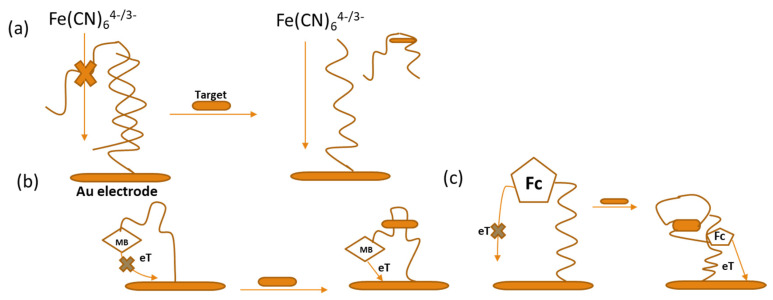
Aptasensors with electrochemical properties. Schematic illustration of the Fe(CN)_6_^4−/3−^ electrochemical aptasensor. (**a**) The structure of the aptamer included a hybridized form with complementary DNA that was fixed onto the gold surface. This aptamer was specifically designed to bind with the target, leading to a decrease in the number of aptamers present on the electrode surface when the target is detected. (**b**) This concept is illustrated in a schematic representation of the electrochemical aptasensor utilizing methylene blue (MB). (**c**) Another schematic diagram shows the Fc-based electrochemical aptasensor. When the target is present, the aptamer folds into a three-way junction that binds the target, which changes the electron transfer (eT) dynamics and results in a higher detected reduction peak. In the presence of the target, the aptamer takes on a constrained hairpin shape, and this change in conformation improves the efficiency of eT between the electrode surface and the ferrocene (Fc) probe [52,53].

Electrochemical methods offer high sensitivity, rapid response, cost effectiveness, ease of use, and selectivity, making them ideal for detecting analytes, even at trace concentrations [48,49]. Different methodologies are applied to the development and assessment of electrochemical sensors including electrochemical impedance spectroscopy (EIS), differential pulse voltammetry (DPV), and cyclic voltammetry (CV) [50]. Amongst these techniques, the usage of CV has been rampant in biosensor studies due to its ability to provide important information about different reduction–oxidation processes during measurements, including reaction reversibility [51]. Additionally, signal transduction involves electroactive reporters like ferrocene, methylene blue (MB), ruthenium complexes, ferrocene-containing polymers, and Fe(CN)_6_^4−/3−^ (Figure 2). In Gothelf’s lab, researchers created an electrochemical aptasensor for the detection of theophylline, where the signal was produced by a redox-active ferrocene (Fc) moiety. This Fc moiety is capable of transferring electrons to the electrode surface using differential pulse voltammetry (DPV) or cyclic voltammetry (CV) [52].

### 3.2. Fluorescence-Based Optical Aptasensors

Fluorescence is one of the most widely used optical techniques in aptasensors [54]. Fluorescent aptasensors can be categorized into two types: labeled and label-free. Labeled aptamers incorporate a fluorophore and a quencher, which are attached to the (A) termini of the aptamer in a molecular beacon configuration, or (B) complementary sequences that hybridize with the aptamer, positioning the fluorophore and quencher in proximity [55]. Upon target recognition, the aptamer undergoes a conformational change, which either enhances or quenches the fluorescence in case (A) or causes the detachment of one or both complementary sequences in case (B). In both cases, the interaction leads to the recovery of the previously quenched fluorescent signal [56]. Label-free fluorescent aptasensors are obtained by hybridization of the aptamer sequence with a perfectly complementary ssDNA or with mismatches that allow the specific intercalation of a dye within the duplex structure. For an aptamer–target complex, the complementary strand will be displaced, thus enabling either signal-on or signal-off sensor configurations. The fluorescence behavior depends on the type of dye used and how the dye’s fluorescence is affected in the presence of a double-stranded DNA molecule [55]. Accordingly, with the use of copper nanoclusters as a fluorescent signal, a new fluorescence sensing method was developed. Colorimetric changes of the AuNPs were converted to polyvinylpyrrolidone (PVP)–copper nanocluster (CuNCs) fluorescence changes through the inner filter effect (IFE). It worked effectively for the detection of aflatoxin B1 (AFB1) present in foodstuffs. Without AFB1, the Apt–AuNPs complex remains in a dispersed state, thus being a very effective quencher of the green fluorescence of PVP-CuNCs. The aptamer then binds to AFB1 when it is introduced, thus displacing AFB1 from the AuNPs. This way, unprotected AuNPs make a color change from red to blue due to NaCl-induced aggregation. The fluorescence signal of the PVP-CuNCs is successively restored as AFB1 concentrations increase, while the fluorescence intensity corresponds directly to the AFB1 concentration. This mechanism is working based on fluorescence recovery and is a sensitive detection system for AFB1 [57].

**Figure 3:**
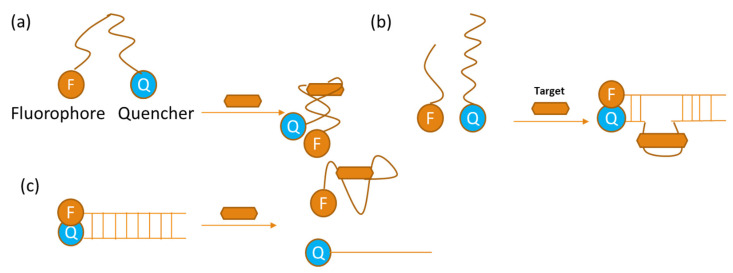
Diagrams showing optical aptasensors that use fluorescence: (**a**) show the most basic type of quenching aptamer beacon, in which the fluorescence is lowered due to the quencher and fluorophore being closer together because of target binding stabilizing the stem. (**b**) provides an example of the assembly aptamer beacon, in which oligomers assemble due to target binding, stabilizing the ternary complex. (**c**) displays the aptamer beacon after disassembly, when target binding causes antisense displacement to occur, increasing fluorescence [52].

Fluorescence detection can be effectively achieved by labeling aptamers with both a quencher and a fluorophore. Using an aptamer-based method, the target was detected effectively through a Forster resonance energy transfer (FRET) signal between fluorescein and 4-(Dimethylamino) azobenzene-4′-carboxylic acid (DABCYL) moieties (Figure 3a). Aptamer beacons act as probes that utilize fluorophore–quencher pairs to identify the presence of the target in solutions. When stimulated, these beacons undergo conformational changes, transitioning into one or more possible states, such as a hairpin or a hybrid structure. More intricate formats have been developed, taking advantage of quaternary structural rearrangements that initiate the assembly and disassembly of aptamers for fluorescence signaling (Figure 3b,c) [52]. 

### 3.3. Colorimetric-Based Optical Aptasensors

Colorimetric assays are simple and cheap, highly sensitive analytical methods that require no expensive instrumentation and complicated techniques. In colorimetric assays, many substances have been used: gold nanoparticles (AuNPs), silver nanoparticles (AgNPs), G-quadruplexes, magnetic particles (MPs), and graphene oxide (GO). The employment of all these materials enhanced the sensitivity and versatility to the assay, hence being effective for quite an immense range of applications [58]. Graphene and the substances produced from it show promising results in sense functioning owing to their outstanding physio-chemical, optical and electrical attributes. This material has a characteristic 2D nanostructure which has a high surface composite enabling the presence of plenty of surface energetic places, which brings about the high association or adherence of the analyte. This characteristic therefore enhances the interaction of the colorimetric sensor with the target molecule, leading to higher sensitivity [59]. Various methods for preparing graphene and its derivatives include mechanical exfoliation, chemical vapor deposition, epitaxial growth, and the classical Hummer’s method for graphene oxide (GO). Modified Hummer’s methods and microwave-assisted techniques are also widely used for GO synthesis [60]. Nanozyme sensors are gaining more and more credit due to their low cost and stability compared with natural enzymes. The advancement of nanomaterials resulted in the demonstration that these materials possess catalytic activity either by themselves or in combination with others. A good example is graphene and its derivatives that show peroxidase-like activity, thus enabling the reaction with 3,3′,5,5′-tetramethylbenzidine (TMB) in the presence of H_2_O_2_ and consequently the color change [61]. Ali et al. demonstrate the use of graphene nanoribbons (GNRs) for colorimetric sensors used to detect the presence of dopamine, which signifies the versatility of these materials in biosensing applications [62]. Enzyme-linked oligonucleotide assay (ELONA) stands for an aptamer-based alternative to enzyme-linked immunosorbent assay (ELISA) which has advantages such as cost-effectiveness, simple design, and the easy labeling of aptamers. In direct ELONA, a single labeled aptamer detects the target, while sandwich ELONA uses two aptamers to reduce the background noise and enhance the specificity. Meanwhile, competitive ELONA can only be used for analytes with limited binding sites. There is also a hybrid approach where some aptamers and some antibodies are used. SELEX, the method of aptamer selection, is an efficient way to gain high specificity, so ELONA is a valuable and versatile tool for diagnostics [63]. 

**Figure 4:**
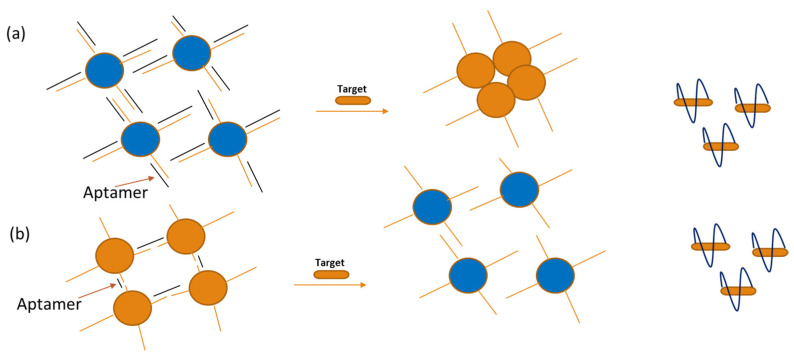
Schematic representations of optical aptasensors using AuNPs: (**a**) shows how target binding causes AuNPs to aggregate and release aptamers. (**b**) shows how target binding causes aptamers to release and AuNPs to disintegrate [52,66].

The application of AuNPs combined with aptamers, so-called nano-aptasensors (NAS), is receiving great interest for a wide range of analytes [64]. The SPRs of AuNPs appear as the collective oscillation of conduction electrons in response to incident light, and intrinsically, AuNPs have a narrow and intensive absorption peak at 520 nm. Regarding this, AuNP-based NASs allow for colored detection owing to a red-to-purple blue shift in the absorption spectrum due to nanoparticle aggregation, which offers a universal, sensitive, selective, and user-friendly detection format. Very recently, NAS systems were developed for the colorimetric identification of antibiotic residues in raw milk samples by overcoming both the pre-analytical challenges and providing a machine-learning algorithm able to improve the sensitivity of the whole analytical process [65]. When the aptamer interacts with the target, the highly negatively charged complementary ssDNA (separated from the aptamer by this contact) is stabilized against aggregation, and this phenomenon is accompanied by a color shift (Figure 4a). The AuNP disaggregation approach, in contrast, was used to identify ATP, cocaine, Pb2+, and K+ (Figure 4b). When the target is present, the aptamer undergoes a conformational change, and as a result, the cross-linked AuNPs are freed from their hybridization under the aptamer and the linker oligonucleotides. These colorimetric methods are advantageous, since they do not need any special equipment and may be seen with the naked eye [52].

## 4. Diagnostic Applications of Aptamers 

Aptamers are emerging as promising alternatives to traditional antibodies in a range of diagnostic applications due to their high affinity and specificity. Like monoclonal antibodies, aptamers can precisely target specific molecular components of their intended targets. They have been effectively used in identifying pathogens, detecting cancer, diagnosing diabetes mellitus, and recognizing chronic respiratory diseases [47].

### 4.1. Pathogens Recognition

Pathogen identification is a prime activity for health and safety in several fields, including environmental surveillance, water quality assessment, clinical diagnostics, and the food industry. The rise of the COVID-19 pandemic has increased interest in activities focusing on the development of accurate, specific, and rapid detection methods. Pathogens include different infectious microorganisms: bacteria, viruses, prions, protozoans, and fungi; they can cause diseases ranging from mild to very severe. Pathogens come from countless sources, including foodborne [67], waterborne [68], and airborne routes of transmission [69], and they enter the human body through different methods of infection [70,71,72]. These infectious agents account for millions of deaths worldwide [73,74,75]. Notable viral pathogens include coronaviruses, influenza viruses, Zika virus, and Ebola virus, while significant bacterial pathogens include *Escherichia coli*, *Staphylococcus aureus*, and *Legionella pneumophila*. Concerns regarding these pathogens differ based on their virulence [76], contagiousness [77], transmission methods [78,79,80], and infectious dose [81]. A prominent example is COVID-19, which has rapidly caused a global pandemic and is continually evolving and enhancing its virulence and transmissibility [82,83]. 

Traditionally, methods for detecting pathogens have been confined to laboratory environments, primarily using microbiological techniques, which are still considered the gold standard. However, isolating pathogens from their natural habitats in a lab setting necessitates specialized tools and skilled personnel. For instance, some fastidious and potentially harmful bacteria require specific growth media and can take over a week to identify microscopically [84]. In situations like COVID-19, identifying the virus in patient samples can often take more than a day, leading to delays in treatment. This underscores the urgent need for rapid, selective, sensitive, and reproducible pathogen detection methods, which highlights the significance of biosensor technology [85].

The transducer component plays a crucial role in biosensor systems, as it converts biochemical reactions into electronic signals. There are various types of transducers available, including mechanical sensors like cantilever biosensors and optical sensors such as surface plasmon resonance (SPR) biosensors. However, electrochemical biosensors (ECBs) are primarily utilized for pathogen detection [86,87]. Transducers, typically made from conducting or semiconducting materials, often function as electrodes. When target pathogens attach to the biorecognition element fixed on the electrode, the biochemical energy produced is converted into electrical energy using electrochemical methods that involve both the electrode and a sample solution. Electrochemical biosensors (ECBs) are highly versatile, allowing for customization to meet various pathogen detection needs. This flexibility can lead to designs that eliminate the need for sample pre-treatment, facilitate in situ detection on different surfaces, use affordable materials for quick results, and support the simultaneous identification of multiple pathogens in contaminated samples. Consequently, ECB technology has significantly enhanced scientific research in pathogen detection across food, water, air, and organic samples for environmental monitoring as well as in identifying pathogens and their toxins in bioterrorism scenarios. Furthermore, advancements in micro- and nanotechnology have enabled the miniaturization of biosensors, which is essential for creating portable devices for pathogen detection [85].

Fluorescence resonance energy transfer (FRET) aptamers have been created as high-throughput screening tools for detecting outer membrane proteins in *Escherichia coli*, which aids in identifying enterotoxigenic *E. coli* (ETEC) K88 [88]. The surface proteins of *Campylobacter jejuni* were also identified with the use of aptamers [89]. Through the bacterial SELEX method, successful detection has been achieved for *Escherichia coli* and other targets, including bacterial proteins [41], *Lactobacillus acidophilus* [90], *Staphylococcus aureus* [91], virulent *Mycobacterium tuberculosis* [92], *Vibrio parahaemolyticus* [93], *Shigella sonnei* [94], and *Campylobacter jejuni* [95]. SELEX-based techniques are also effective in creating molecular probes for identifying viral infections, such as those caused by herpes simplex virus [96], hepatitis C and B viruses [97,98], human immunodeficiency virus [99], avian influenza virus [100], and the coronavirus responsible for Severe Acute Respiratory Syndrome (SARS) [101]. Moreover, SELEX has shown great success in developing aptamers for detecting various parasitic organisms. Specifically, it has been employed to obtain aptamers that target several parasites, including *Trypanosoma* spp. [102], *Leishmania* spp. [103], *Plasmodium* spp. [104,105,106,107,108,109,110,111,112,113], *Cryptosporidium parvum* [114], and *Entamoeba histolytica* [115]. Ospina-Villa et al. gave a detailed review of recent progress related to the use of aptamers in diagnosing protozoan parasites [116].

### 4.2. Cancer Recognition

RNA-based biosensor technology is still in its early stages, but its specificity and accessibility have attracted considerable attention for cancer diagnosis and prognosis. Current research is concentrating on miRNA-based biosensors that use nanoparticles as potential diagnostic tools for cancer [117,118]. Moreover, biosensors that utilize aptamers have demonstrated potential in diagnosing various cancers, including lung [119], ovarian [120], and other cancers [121]. Lung cancer, the most prevalent cancer worldwide, can now be detected through various nucleic acid-based (NAB) methods. Researchers are working on biosensors to identify genes and biomarkers associated with lung cancer, such as TP53 and K-RAS. A newly developed DNA biosensor has been shown to detect single nucleotide polymorphisms (SNPs) in the TP53 gene, using a synthetic DNA probe that can hybridize with its mutant form, as identified through surface plasmon resonance (SPR) and quartz crystal microbalance (QCM) analysis [122]. Several studies have pinpointed various biomarkers for bladder cancer detection using NABs [123]. For the early detection of bladder cancer biomarkers, a sensitive nucleic acid biosensor has been created, employing a silicon resonator as an optical sensor that targets FGFR3 (fibroblast growth factor receptor 3) and HRAS (Harvey RAS) in urine samples. This biosensor uses DNA probes specifically designed to bind to their mutant FGFR3 and HRAS targets [124].

Breast cancer, a significant and serious malignancy that affects women (accounting for nearly 25% of all cancers), presents a poor prognosis [125]. This type of cancer is driven by several hormonal imbalances (e.g., HER2, estrogen receptor, progesterone receptor, ki-67, and MUC1) and abnormalities in signaling pathways (e.g., p53 and RAS pathways) [126]. Recently, a peptide nucleic acid (PNA)-based biosensor that employs a fluorescent assay has shown promise as a tool for detecting and quantifying HER2 oncogene expression, offering excellent specificity and sensitivity in distinguishing between wild-type and mutant targets [127]. Additionally, a new electrochemical aptamer-based biosensor has effectively identified breast cancer cells that overexpress MUC1 proteins by using a sandwich formation method with MUC1-specific aptamer assays [128]. Cervical cancer, another prevalent cancer in women primarily associated with human papillomavirus (HPV), has been addressed by a highly sensitive and selective leaky surface acoustic wave (LSAW) PNA biosensor designed for detecting HPV genomic DNA in clinical samples [129,130]. The advancement of NABs with multiplex detection capabilities holds great promise for providing rapid and cost-effective cancer diagnostics.

Leukemia is a well-researched cancer marked by the abnormal growth of blood cells that originate from hematopoietic tissues like the bone marrow [131]. To effectively treat this condition, precise and sensitive diagnostic methods are essential. Current techniques for identifying leukemia cells include flow cytometry, polymerase chain reaction, and fluorescence measurements [132]. As a result, a sensitive and quantitative biosensor with point-of-care capabilities has been proposed for both diagnosis and treatment [133]. This device merges the optical properties of gold nanoparticles with the benefits of aptamers—single-stranded oligonucleotides that specifically bind to target molecules—providing high specificity, stability, and ease of production compared to traditional molecular probes [134]. By using gold nanoparticles coated with aptamers, the biosensor enables a cost-effective qualitative and quantitative detection of cancer cells in the bloodstream with potential future applications for identifying various cancer types through different aptamers. Overall, the advantages of aptamers indicate a promising therapeutic potential in cancer treatment, including the ability to modulate immune receptors in lymphocytes, which can help counteract immune suppression or boost the immune response against tumors [88,135,136,137]. Furthermore, aptamers can assist in the targeted delivery of anticancer agents, such as chemotherapeutic drugs or small interfering RNAs (siRNAs), directly into cancer cells.

### 4.3. Diabetes Mellitus Diagnosis 

Diabetes mellitus is the most prevalent metabolic disorder, and it is marked by high glucose levels. To accurately diagnose and manage diabetes, the effective detection and monitoring of blood glucose levels is essential. The glucose detection biosensor technology market accounts for nearly 85% of the global biosensor market [138]. However, there are only a few published nucleic acid-based biosensors (NABs) for detecting biomarkers and miRNA levels related to diabetes. Personal glucose meters (PGMs) that utilize DNA have been developed for blood glucose monitoring, employing enzymatic reactions to detect 40 pM DNA without relying on PCR. This quantification method involves coupling cDNA with target analyte DNA through invertase [139]. Additionally, changes in miRNA expression levels have been associated with diabetes. This method uses nanosized graphene oxide as a quencher to inhibit the fluorescence of PNA probes with fluorescence being restored upon the binding of PNA to the target miRNA [140]. For type 2 diabetes, a common metabolic disorder associated with insulin resistance, an aptamer-based biosensor has been created for the quick detection of Retinol Binding Protein 4 (RBP4) levels in serum samples. This biosensor was designed by immobilizing RBP4-specific aptamers on a gold chip and employing the SPR detection method [141].

### 4.4. Chronic Respiratory Diseases 

Chronic diseases (CDs), defined as prolonged infections with persistent symptoms, are among the leading causes of death globally, accounting for 70% of overall healthcare expenditure [142,143]. According to the World Health Organization, CDs pose a significant health risk, especially to the older as well as younger populations, and they are more lethal in developing regions due to fewer resources being available [144]. Although appropriate treatment can enhance the management of CD and slow disease progression [145], early diagnosis—one of the backbones of effective care—has been impeded by prevalent clinical practices that depend on slow, expensive diagnostic procedures. The additional time required to attain diagnosis further complicates challenges related to healthcare resource allocation and coordination, leading to higher financial burdens [146].

Recent developments in biosensors utilizing aptamers have shown the capability to tackle these issues. As an illustration, a sensor of the electrochemical type was created employing crude methods to identify lung cancer [147]. The sensor, with the LC-18 aptamer, achieved an impressive detection limit of 0.023 ng/mL by incorporating beads in the system. In addition, a label-free lab-on-chip (LoC) electrochemical sensor has been developed for quantifying *Mycobacterium tuberculosis* DNA extracted from clinical samples [148]. The sensor design, using carbon nanotubes and ferrocene, significantly improved the LOD by enabling higher DNA-capture rates and thus lowered the LOD from picomolar (pM) to femtomolar (FM) levels. This increased sensitivity, along with a dynamic range of 0.1 FM to 1 pM, represents a significant enhancement compared to conventional microelectrodes, which commonly range from 1 pM to 100 nM. These developments are important for the accurate quantification of pathogenic analytes that manifest wide variability in clinical samples. The direct genomic detection of pathogens eliminates the need for amplification and provides a more robust means of pathogen quantification. The addition of a sample processing module capable of enabling the analysis of unprocessed samples would go a long way to enhance the appeal of point-of-care (POC) testing.

Non-communicable diseases (NCDs) account for 71% of deaths worldwide each year, highlighting the urgent need for prompt diagnosis to facilitate efficient treatment, prevention, and management. Among the most essential ASSURED (affordable, sensitive, specific, user-friendly, rapid and robust, equipment-free, deliverable) criteria for diagnostics pertaining to NCDs are user-friendliness, rapidity, and cost-effectiveness. Biochemical tests (such as glucose and hemoglobin) and biological tests (like electrical, neural and muscular tests) are commonly used in the diagnosis of non-communicable diseases (NCDs). These tests typically require laboratory facilities and considerable, complex equipment resources that are often unavailable in resource-poor settings. Additionally, challenges of inaccessible laboratories, poor sample transportation, and maintaining sample integrity all severely limit the process of diagnosis and have a role in the increased cost burdens. Such habits have brought about a paradigm shift in the field of NCD diagnostics from a centralized laboratory setting to point-of-care solutions. In the last decade, great strides have been made in POC diagnostics, as can be exemplified by advancements in handheld glucometer devices for diabetes management, enabling rapid testing and even self-monitoring by patients. These POC devices, including the examples of urine acid hydrolysis-derived biosensors and test strips, have revolutionized the diagnostics by increasing patients’ willingness to be tested and have enabled clinical decisions to be made rapidly. This transition, from a centralized diagnostics to more accessible POC tools, holds the greatest potential to improve both disease management and healthcare delivery—especially in resource-constrained environments [149].

## 5. Conclusions

Aptamer-based biosensors have been shown to be the best alternative to individual diagnostics with regard to their real requirements in the personalized settings. Due to their high specificity, stability as well as flexibility, they are very helpful in identifying disease biomarkers such as cancer, diabetes, and chronic respiratory disorders. The SELEX method has also yielded the further development of aptamers, which now can fabricate biosensors with much higher sensitivity and accuracy.

The paper gives instances of applications of aptasensors in areas like detecting SARS-CoV-2, the treatment of geographic atrophy with Avacincaptad pegol, and monitoring chronic diseases. However, there are some challenges like scalability, stability under various conditions, and integration into clinical workflows. The advancements in electrochemical and optical aptasensors have shown that they can fulfill the requirements of ASSURED criteria. Nevertheless, the restricted robustness in harsh environments and the need for more scalable manufacturing methods are other issues that limit their widespread clinical usage.

Future efforts should prioritize expanding the range of detectable biomarkers, improving device miniaturization for portable use, and enhancing robustness for operation in various settings. Addressing these gaps will enable aptamer-based diagnostics to fully realize their transformative potential in global healthcare. 

## Figures and Tables

**Table 1:** Aptamers and antibodies [23,26,27].

Criteria	Aptamers	Monoclonal Antibodies
Molecular weight/size	Molecular weight range: 6–30 kDa (20–100 nucleotides) the size of 2 nm	150–180 kDa, high molecular weight 15 nm or so
Possible targets	A wide variety of substances, including as organic molecules, ions, nucleic acids, carbohydrates, amino acids peptides, cells, toxins, organic compounds and antibiotics might serve as possible targets	only immune-stimulating substances, low toxins
Creation and production	In vitro SELEX 2 to 8 weeks, although with HTS-SELEX, it may only take hours. Minimal chance of contamination. Possibility of mass manufacture. It is possible to change the environmental selection criteria	In vivo biological system. Biological system used in vivo around 6 months or more. Possible contaminant brought on by the use of animal products or cells. It is impossible to produce on a large scale without sacrificing the final product’s quality. The ambient factors must be compatible with the physiological setting.
Reproducibility	No batch-to-batch fluctuation and high reproducibility	Large batch-to-batch variations
Maintenance	Enduring quality and strength able to be lyophilized, conveyed conveniently, and kept at room temperature	A short shelf life, instability For storage and transit, it must be chilled. Sensitive
Physical and thermal stability	Tolerant of extreme temperatures. May be denatured reversibly. Reusable	Temperature-sensitive (even at 37 °C or room temperature). Vulnerable to permanent denaturation. Single use
Chemical Modification	Numerous site-specific chemical alterations that can be conveniently carried out during synthesis or before selection	Limited, non-specific, and inconsistent chemical changes
Immunogenicity	None or low	High
Pharmacokinetics (nuclease decay, kidney filtration, and tissue absorption)	Highly effective biological compartment entrance, sensitive to renal filtration and nucleases Pharmacokinetic qualities with a short half-life can be enhanced	Restricted access to many biological compartments, resistant to nucleases, and incapable of undergoing renal filtration. Long circulation half-lives make pharmacokinetic changes difficult
Specific antidote	Yes	No

## Data Availability

Not applicable.
